# Strong correlation between phantomless and inline phantom-based densitometric calibration of vertebral properties from CT scans of healthy volunteers

**DOI:** 10.3389/fbioe.2026.1781532

**Published:** 2026-05-29

**Authors:** Fiona G. Gibson, Zichu Ding, Margaret A. Paggiosi, Catherine Handforth, Janet E. Brown, Xinshan Li, Enrico Dall’Ara, Stefaan W. Verbruggen

**Affiliations:** 1 School of Mechanical, Aerospace and Civil Engineering, The University of Sheffield, Sheffield, United Kingdom; 2 Insigneo Institute, The University of Sheffield, Sheffield, United Kingdom; 3 Centre for Bioengineering, School of Engineering and Materials Science, Queen Mary University of London, London, United Kingdom; 4 Division of Clinical Medicine, School of Medicine and Population Health, The University of Sheffield, Sheffield, United Kingdom; 5 Leeds Teaching Hospitals NHS Trust, Leeds, United Kingdom; 6 Digital Environment Research Institute, Queen Mary University of London, London, United Kingdom

**Keywords:** BMD, bone mineral density, CT scan, phantomless calibration, vertebral biomechanics

## Abstract

Phantom calibration is currently the gold standard for calibrating CT scans and for calculating material properties of dense tissues for computational models. However, in Oncology departments and low-resource settings, it is not routine to include a calibration phantom within the scanning protocol. Therefore, retrospective scan datasets are challenging to calibrate for biomechanical investigations, precluding detailed measurements of material and mechanical properties. In this study, we compared the results from a phantomless calibration technique, where the density within each scan was independently calibrated based on known tissue densities captured within each scan (e.g., air), with those from a traditional inline phantom calibration. To do so we used scans from a cohort of healthy volunteers from the control arm of a clinical trial dataset (ANTELOPE) in which inline calibration phantoms were included. We found that, when selecting air and the aorta as regions for calibration within individual CT scans, a strong individual-specific correlation existed between bone mineral density measured in the phantomless and phantom calibrations. This indicates that the phantomless calibration method can be a useful and reliable tool for quantifying the densitometric material properties of healthy human vertebrae, and provides the opportunity for further analysis of spinal CT scans in either retrospective datasets or in low-resource clinical settings.

## 1 Introduction

To assess the patient’s risk of vertebral fracture in clinics, two imaging modalities are typically deployed, Dual Energy X-rays Absorptiometry (DXA) and Quantitative Computed Tomography (QCT). At present, DXA remains the gold standard in the clinic due to ease of use, speed, cost and the low radiation exposure (5–20 µSv by DXA vs. 60–90 µSv by QCT) (Kabayel, 2016). QCT provides images obtained from a CT scanner, where either an inline or offline (retrospective, same scanner and scanning protocol) calibration phantom could also be scanned. Calibration phantoms are used to convert the image’s Hounsfield units into equivalent bone mineral density (BMD) through a set of equations.

The role of CT technology has grown extensively due to its effectiveness in skeletal assessment for diagnosis and continuous monitoring of cancers (Duvauferrier et al., 2013). This imaging technique provides essential information for assessing spinal stability by allowing for the identification of osteopenia, lytic lesions, soft-tissue involvement and fractures (Mahnken et al., 2002). The main concern with CT use is the exposure to significantly higher doses of radiation in comparison to standard radiographs (Winterbottom and Shaw, 2009). Despite this, evidence suggests that low-dose whole body CT is effective in producing high resolution images that provide the information necessary for assessing spinal stability (Gleeson et al., 2009).

The densitometric calibration of QCT images, required to compare data across different types of scanners and protocols (Pickhardt et al., 2013; Carpenter et al., 2014), usually utilises an external phantom; however, routine QCT scans are often conducted without a densitometric calibration phantom as its usage increases the logistical burden of clinical imaging (Lee et al., 2017). To account for this, numerous methods for phantomless calibration have been developed (Michalski et al., 2020; Bartenschlager et al., 2022). One approach is to pre-calibrate the scanner using either DXA measurements or a calibration phantom and apply this general pre-calibration factor to prospective QCT scans (Budoff et al., 2013; Pickhardt et al., 2015). Even though this is an improvement from not performing any densitometric calibration, it does not consider the patient-specific differences as well as scanner and protocol changes.

The most widely used phantomless approach is to utilise internal tissues as the reference materials. The choice of tissues has been varied and depend on scan location, with some authors chose air, fat and blood (Lee et al., 2017; Schwaiger et al., 2017), while others used fat and muscle (Weaver et al., 2015; Saffarzadeh et al., 2016) and showed similar results. In particular, Bartenschlager et al. compared different combinations of two internal tissues when calibrating for vertebrae BMD, reporting the lowest error for any combination with air (<5%), particularly air and blood and the highest errors arising when using muscle in the combination (Bartenschlager et al., 2022). These authors also concluded that the use of different CT scanners did not result in significant differences in calibration outcome. However, as their study was conducted in female patients with osteoporosis, it remains to be demonstrated whether these same settings can be generalised to apply to healthy or male participants. Additionally, their study was carried out on lumbar vertebrae, and as such it remains unclear if these findings apply to other spinal sites.

Therefore, the aim of this study was to investigate whether phantomless densitometric calibration of QCT scans can assess bone mineral density in the vertebra of healthy participants similarly to current gold standard approaches based on phantom calibration. Furthermore, we aimed to assess whether the accuracy holds over multiple timepoints and multiple scanning systems. We explored this by applying these techniques to healthy male volunteers in the control cohort of a clinical trial. Furthermore, this allowed us to compare these calibration methods across multiple time points (baseline and follow-up scans) and with two different scanners.

## 2 Materials and methods

### 2.1 Participant data

The dataset analysed in this study is a time series QCT dataset from the ANTELOPE clinical trial (Gibson et al., 2025). Full details of the ANTELOPE trial, which investigated the skeletal effects of androgen deprivation therapy on prostate cancer patients, and demographics of the participants have been described elsewhere (Handforth et al., 2024; Gibson et al., 2025). Ethical approval was obtained from the South Yorkshire Research Ethics Committee (IRAS ID 206171). As the focus of this study was to identify the applicability of phantomless calibration in healthy bone, the 25 male healthy volunteers in the trial’s control group were taken as the cohort for this study. While the trial investigated the effects of androgen deprivation therapy in prostate cancer patients (i.e., the treatment group), none of these cancer patients were included in this study. Moreover, there was no presence of vertebral fracture in any of the participants’ T12 vertebra assessed in this study. A total of 50 scans was analysed, with two scans for each of the 25 participants at baseline (0 months) and follow-up (12 months). Participant details are shown in Table 1.

**TABLE 1:** Participant demographics data for the 25 male healthy volunteer in the control group of the ANTELOPE clinical trial including age, height, and BMI.

Participant ID	Age (years)	Height (cm)	BMI (kg/m^2^)
C01	74	192.3	31.4
C02	76	169.0	22.3
C03	77	180.1	30.1
C04	73	173.3	26.2
C05	78	170.4	26.9
C06	68	182.8	27.1
C08	53	184.4	26.9
C09	63	188.1	29.6
C10	70	161.0	29.3
C11	79	175.0	30.7
C12	78	179.0	32.1
C14	74	168.2	26.8
C15	80	175.2	26.3
C16	82	169.0	29.7
C17	78	169.0	24.9
C19	75	180.8	34.9
C20	73	178.0	24.2
C21	78	159.8	22.7
C23	77	163.2	31.8
C24	73	184.5	23.9
C25	71	182.0	24.9
C26	71	173.0	20.4
C30	75	181.0	25.5
C31	71	171.0	27.6
C32	64	171.7	23.5
Average (±SD)	73 ±6	175.9 ± 8.0	27.1 ± 3.5

### 2.2 Phantom calibration

The QCT scan protocol for this trial included a solid inline calibration phantom (Image Analysis, Inc., Columbia, KY, USA) containing rods of 0, 0.075, and 0.15 g/cm^3^ equivalent concentration of calcium hydroxyapatite. This was carried out as per manufacturer instructions, and following well-established standards described in depth elsewhere (Burghardt et al., 2010). The first cohort from 2017 (29 participants) was scanned at baseline using the GE LightSpeed VCT (GE Healthcare, Milwaukee, WI) in the radiology department at the Northern General Hospital, Sheffield, UK, whilst the follow-up scans in 2018 along with all second cohort scans (25 participants who completed all assessments) were scanned using the Toshiba Aquilion ONE (Toshiba Medical Systems, Tokyo, Japan) at the same hospital. Quality assurance was performed once per month using a Mindways phantom (Mindways Software, Inc., Austin, TX, USA) on both scanners. All scans were performed in the anteroposterior position, using the same noise index. The QCT protocol included a single scan from the cranial endplate of the T12 vertebra to the T12/L1 margin. For the GE scanner, the tube voltage was 120 kV and the mean tube current was set at 360 mA, with a voxel size of 0.937 × 0.937 × 0.625 mm^3^. For the Toshiba scanner, the tube voltage was also 120 kV, the mean tube current was set at 250 mA and a voxel size of 0.976 × 0.976 × 0.5 mm^3^.

**FIGURE 1:**
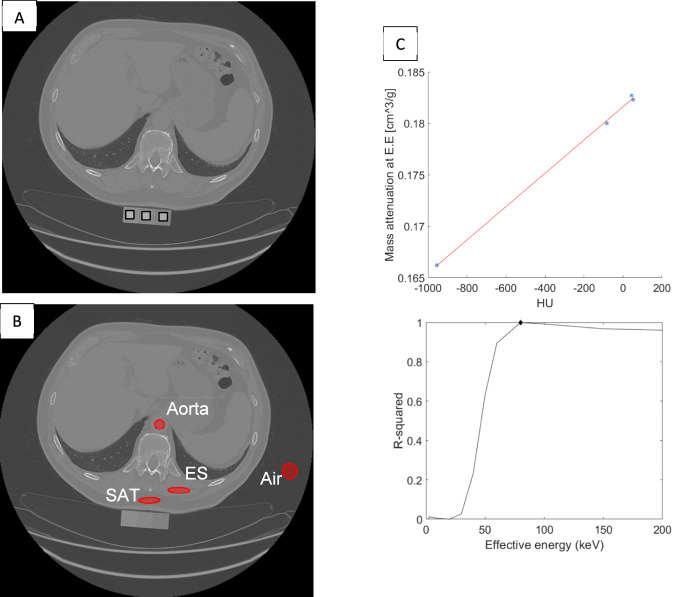
Density calibration methods for quantitative CT analysis. **(A)** Phantom-based calibration uses the phantom. Each calibration rod is sampled from the image to determine linear conversion between HUs and equivalent density (black square boxes). **(B)** In scan tissues of reference (adipose (SAT), air, blood (aorta), and skeletal muscle (ES)) are sampled adjacent to the bone of interest for internal calibration. **(C)** HUs and mass attenuation coefficients for each tissue (circle data points) are correlated by iterating at each effective energy (EE). Scan effective energy is determined by maximizing the coefficient of determination across all effective energies (black diamond).

The densitometric calibration was computed using a standard approach, which assumes a linear relationship between the average Hounsfield units (HU) and the known equivalent mean values of equivalent BMD of each rod. To do so, one region of interest (ROI) (black square boxes, Figure 1A) was defined manually within each insertion of the phantom (ImageJ) (Rasband, 1997; Schneider et al., 2012). ImageJ is a widely-used, open-source image processing and analysis program originally developed at the National Institutes of Health (NIH), written in Java to serve as a highly flexible and standard tool in biomedical research (Rasband, 1997; Schneider et al., 2012). The ROIs were defined as square regions centred within each calibration rod with length equal to half the edge length of the rod (12.5 mm). For each of the three rods, mean HU values over the same 10 slices were used to perform the linear regression analysis for calibration, Equation 1.

$${\rho_{QCT}}^{p}=a+bHU$$ (1)

where ${\rho_{QCT}}^{p}$ represents the QCT equivalent BMD calculated using the calibration phantom, *HU* represents the Hounsfield unit values of the densitometric calibration law and a and b are constants from the linear regression analysis performed. This equation was applied to estimate the equivalent BMD in each voxel.

This equation can then be applied to estimate the BMD of the bone (Equations 2, 3). From this equation, the apparent density and the elastic modulus of the tissue are calculated using the following relationships. The density determined by CT (ρ_QCT_) and ashing density (ρ_ash_) is highly correlated (Schileo et al., 2008), therefore it is assumed that *ρ_QCT_ = ρ_ash_* (Schileo et al., 2008).

$$\rho_{ash}=\rho_{app}\times 0.6\;\left[g/{cm}^{3}\right]$$ (2)

where ρ_QCT_ is the QCT equivalent BMD and ρ_app_ is the apparent density (Morgan et al., 2003).

$$E=4730{\rho_{app}}^{1.56}\;\left[MPa\right]$$ (3)

where E defines the elastic modulus as a function of the apparent density, ρ_app_. This allows mechanical properties, reliant upon the densitometric conversion, to be calculated as previously (Gibson et al., 2025).

For the assessment of BMD, from each QCT an ellipse shaped ROI was identified (ImageJ) in the anterior most region of the vertebral body. The ellipse was identified by creating a circular region of interest in the vertebral body, ensuring the cortical portion was included. HU values within the ROI for all slices were converted into BMD using the densitometric calibration identified as described above. To capture the integral BMD (g/cm^3^), the FE software Ansys Workbench (2021R1) was used to select a ROI for all the elements in each vertebral body after segmentation and reconstruction as described elsewhere (Gibson et al., 2025), excluding the posterior elements and processes, incorporating both the cortical and trabecular regions. The integral BMD was then calculated as the sum of the individual element’s bone mineral content (element BMD multiplied by element volume) divided by the total volume of the vertebral body ROI.

### 2.3 Phantomless calibration

To conduct the phantomless calibration, a combination of internal materials (IM) was used. In depth explanations of phantomless calibrations are available elsewhere (Lee et al., 2017). Briefly, from each scan, tissue ROIs for subcutaneous adipose tissue (SAT), air, aortic blood, and the erector spinae skeletal muscle (ES) were manually sampled from the scan field-of-view, as depicted in Figure 1B. To reduce influence of variations in tissue HUs across the scan field-of-view, the ROIs were placed adjacent to the bones of interest (T12 vertebra) for each tissue, and the mean HUs were determined from the tissue sample aggregated histograms of ten 2D slices, providing a compromise between the size of the ROI and size of the anatomical feature of interest. Using mass absorption coefficients (Table 2) obtained from the National Institute of Standards and Technology (www.nist.gov National Institute of Standards and Technology, NISTIR 4999), the scan effective energy was estimated by iteratively correlating the ROI-specified HUs and corresponding mass absorption coefficient at each energy level and maximizing the coefficient of determination (Millner et al., 1978), as shown in Figure 1C. This calibration for scan effective energy is necessary, as different local geometry and material properties affect the local attenuation of the X-ray beam, and this procedure allows more accurate correlation of the local HUs to the material properties (Millner et al., 1978). Additionally, the coefficient of determination can vary greatly at different effective energies, as demonstrated in Figure 1D, and therefore emphasises the importance of maximising this coefficient.

**TABLE 2:** Mass densities of internal calibration materials obtained from the National Institute of Standards and Technology (NIST) database.

Material	Density (g/cm^3^)
Blood	1.06
Air	1.205 × 10^−3^
ES	1.05
SAT	0.95

ES, erector spinae muscle, SAT, subcutaneous adipose tissue.

For compounds such as hydroxyapatite (Ca10(PO4)6(OH)2) that are not tabulated in NIST, mass absorption coefficients can be calculated if the atomic mass fractions and the mass densities are known. When a compound like hydroxyapatite (HA), is not listed in standard databases like NIST, its mass absorption coefficient must be calculated using the mixture rule. First, the weight fraction of each element (Ca, P, O, H) was calculated by dividing its total atomic mass in the formula by the total molecular weight of HA. Once the scan effective energy was determined for the scan, the mass absorption coefficients, equivalent density and measured HU values for each material were used in a two-component mass fraction model (Genant and Boyd, 1977) to calculate the associated calibration equation, Equation 4.

$${\rho_{QCT}}^{pl}=\frac{{\left(\frac{\mu }{\rho }\right)}_{1}\rho_{1}\frac{HU-{CT}_{2}}{{CT}_{1}-{CT}_{2}}+{\left(\frac{\mu }{\rho }\right)}_{2}\rho_{2}\frac{HU-{CT}_{1}}{{CT}_{2}-{CT}_{1}}-{\left(\frac{\mu }{\rho }\right)}_{w}\rho_{w}}{{\left(\frac{\mu }{\rho }\right)}_{HA}-{\left(\frac{\mu }{\rho }\right)}_{w}\frac{\rho_{w}}{\rho_{HA}}}$$ (4)

where ${\rho_{QCT}}^{pl}$ represents the BMD calculated using the phantomless calibration method, *HU* represents the Hounsfield unit values of the densitometric calibration law, ${CT}_{1}$ and ${CT}_{2}$ represent the averaged grey value of each internal material, $\rho_{1}$ and $\rho_{2}$ represent the density of each internal material, $\rho_{w}$ and $\rho_{HA}$ represent the density of water and hydroxyapatite respectively and ${\left(\frac{\mu }{\rho }\right)}_{1}$ and ${\left(\frac{\mu }{\rho }\right)}_{2}$ represent the mass absorption coefficient for the internal material, ${\left(\frac{\mu }{\rho }\right)}_{w}$ and ${\left(\frac{\mu }{\rho }\right)}_{HA}$ represent the mass absorption coefficients for water and hydroxyapatite respectively.

The total ${\left(\frac{\mu }{\rho }\right)}_{HA}$ is the sum of the individual coefficients of its constituent elements, each multiplied by its respective mass fraction. Because $\left(\frac{\mu }{\rho }\right)$ varies with X-ray energy, the calculation must be performed at the scan effective energy (the monoenergetic equivalent of the polychromatic X-ray beam). The Genant and Boyd (1977) model assumes that any given volume (voxel) in the bone can be represented as a mixture of two primary components: bone mineral (hydroxyapatite) and soft tissue (represented as water). The terms involving, for example, $HU-{CT}_{1}$ , represent a linear interpolation between two known internal reference materials that have been measured in HU values (e.g., fat), and by combining with the density values, the terms essentially convert the abstract HU values back into physical attenuation values based on the specific scanner’s performance during that scan. The term $-{\left(\frac{\mu }{\rho }\right)}_{w}\rho_{w}$ subtracts the expected attenuation of water. This isolates the “excess” attenuation caused by the presence of bone mineral. Thus the final calculated value ${\rho_{QCT}}^{pl}$ provides a quantitative measure of BMD that is corrected for the specific energy of the CT scan and the patient’s own internal tissue characteristics, allowing for “phantomless” screening using routine clinical scans.

The two calculated BMD values were compared directly in order to estimate the accuracy errors for each of the 50 scans, with measured trabecular CT values of the T12 vertebra converted to BMD using both the phantom and phantomless calibration equations. Then for each scan, the difference (ΔBMDs) between the phantom based and phantomless approaches was determined.

### 2.4 Statistical analysis

The linear regression analysis was used to compare the values of BMD calculated for each vertebra at both time points using the phantom and the phantomless calibration methods. For statistically significant regressions (p < 0.05) the Spearman’s correlation coefficient (r) was reported. An *a priori* power analysis was conducted to determine the required sample size for a Spearman’s rank-order correlation. The calculation was based on a two-tailed test, an alpha (*α*) level of 0.5, and 80% power (1-*β*) of 0.80. To achieve these parameters to detect a strong effect size (*ρ* ≈ 0.62), an *n* = 20 is required. This sample size is sufficient to identify meaningful monotonic relationships while allowing for non-parametric nature of the data. Steiger’s z-test was used to compare the statical significance of differences (z) between correlations established using regression analyses.

## 3 Results

**FIGURE 2:**
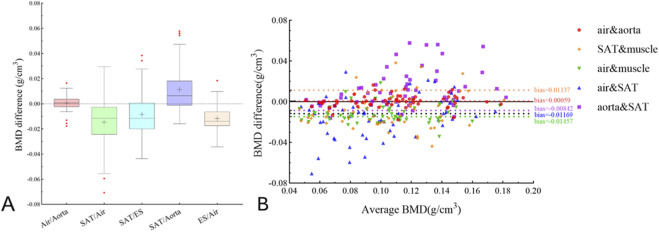
**(A)** BMD differences between phantomless and phantom-based calibration for five different internal materials (IM) combinations. All scans were included in each analysis, and top and bottom horizontal borders of each box indicate the 25th and 75th percentiles with their distance representing the interquartile range (IR), the black line shows the median. Red points outside the dashed lines (Whisker) are outliers with values >1.5 × IR. **(B)** Test for systematic bias in the compared datasets.

The systematic bias and random variability for BMD difference between phantom based and phantomless calibrations are shown in Figure 2. Here, each datapoint represents the average BMD for an individual scan as calculated if calibrated with the indicated combination of internal materials. In Figure 2A, each boxplot contains 50 values for a given internal-material combination, while in Figure 2B, each point represents one scan, where the y-axis is the difference between phantomless and phantom-based mean T12 BMD, and the x-axis is the average of those two scan-level mean BMD values. The largest biases were found for the combination of air and SAT (−0.0146 g/cm^3^, underestimation) and aorta and SAT (0.0114 g/cm^3^, overestimation). The air and aorta combination demonstrated the highest accuracy, with a negligible bias of 0.0006 g/cm^3^ and the lowest variability (SD = 0.006 g/cm^3^). These correspond to relative errors of 0.6% (air/aorta), 10.6% (aorta/SAT), −13.6% (air/SAT), −10.9% (air/muscle), and −7.9% (SAT/muscle).

**FIGURE 3:**
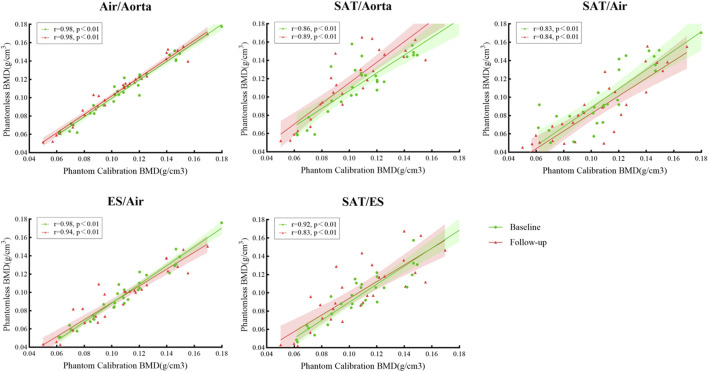
Linear regressions between the phantom and phantomless BMD for all internal material combinations, and for each group of baseline and follow-up scans.

Figure 3 reports the linear regressions between the phantom based and phantomless BMD values for all internal materials combinations. It can be seen that all regression analyses were statistically significant (p < 0.01 in all cases) and that the maximum variation between baseline and follow-up datasets was 0.09 (for SAT/ES). The least variation in regression between baseline and follow-up data was for Air/Aorta, with an r = 0.98 for both datasets.

Here we found that calibration using a combination of the air in the scan, and the blood inside the aorta, provided the strongest correlation with standard phantom calibration (r = 0.98, p < 0.01). Additionally, using Steiger’s z-test to compare the statical significance of differences between correlations (Table 3), we found that calibration using air and aortic blood also outperformed the second-best method (air and muscle) (Steiger’s z = 2.90, p < 0.05). This is also inherently sensible, as the densities of air and blood are well characterised and, compared to the density of a mineralised tissue like bone, do not vary significantly from participant to participant.

**TABLE 3:** Comparison of regression equations between the best performer air/aorta and each of the other internal calibration materials using Steiger’s z-test.

Test	Air/Aorta vs. SAT/Aorta	Air/Aorta vs. SAT/Air	Air/Aorta vs. ES/Air	Air/Aorta vs. SAT/ES
z	7.5085	7.5512	2.8956	6.9338
p-value	<0.0001	<0.0001	0.0038	<0.0001

**FIGURE 4:**
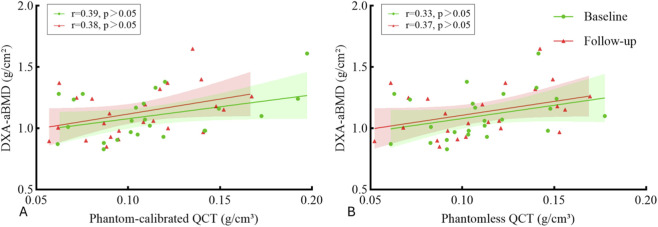
Linear regressions show similar relationships between areal BMD (aBMD) values recorded by DXA and **(A)** phantom-calibrated or **(B)** phantomless volumetric BMD (BMD), using air/aorta as internal material combinations, and for each group of baseline and follow-up scans.

Lastly, to investigate the effectiveness of phantomless calibration in the clinic, we directly compared both calibration methods to DXA measurements of areal BMD from the same patients at the relevant time point within the ANTELOPE study (Figure 4). As we have found previously (Gibson et al., 2025), areal BMD values generated by DXA are not highly predictive of volumetric BMD, and therefore fracture risk. The lack of volumetric detail in DXA scans is one of the disadvantages to what is an otherwise widely available and useful technique for patient stratification. However, it is noteworthy that in a regression analysis DXA was equally predictive of QCT volumetric BMD values regardless of whether phantom- or phantomless-calibration was used. This further highlights that phantomless calibration provides equal clinical utility to the current gold-standard phantom calibration method.

## 4 Discussion and conclusion

This study compared between phantomless and phantom based calibration, and evaluated the use of different combinations of internal materials for the phantomless approach. The combination of aorta and air for calibrating the CT scans yielded the most similar volumetric BMD with a 0.41% difference when compared with the gold standard phantom method (high correlation of 0.98), and equally as predictive as phantom-calibration when compared against DXA measurements. These findings confirmed previous work showing that the phantomless calibration method is a useful and reliable tool for quantifying the material and mechanical properties of human vertebrae (Bartenschlager et al., 2022). As expected, this study confirmed that phantomless calibration is equally accurate in male healthy volunteers in clinical trial conditions, indicating that phantomless techniques may be applied more broadly, and providing the opportunity for further analysis of spinal CT scans in either retrospective datasets or in low-resource clinical settings.

An important limitation to note is that, while this study analyses healthy volunteers from the control arm of a clinical trial, the participants are older and not representative of the general population. It would indeed be useful to expand this method to study a wider range of age and BMI. Nonetheless, as previous studies have investigated scans of osteoporotic women, it is a finding of note that our study gives strong confidence that these methods are robust in an equivalent elderly male population.

A number of previous studies have demonstrated the potential of phantomless calibration for densitometric analysis of bones using QCT scans. Early work on vertebrae found that it was possible to apply phantomless calibration to the lumbar spine scans of patients injured in traffic accidents, showing excellent agreement with phantom calibration in these patients (Weaver et al., 2015; Saffarzadeh et al., 2016). Similarly, a study comparing phantomless predictions in prostate cancer patients found strong agreement in vertebral and femoral fracture risk prediction in these patients (Schwaiger et al., 2017). Further analysis by this group showed very low inter-operator variability with phantomless calibration using femur QCT scans of patients, indicating it is a reliable form of calibration (Lee et al., 2017). Most recently, Bartenschlager et al. used a retrospective QCT database of post-menopausal women to show that phantomless calibration was highly correlated with phantom based method, and that the best internal materials to use were values measured from the aorta and air (Bartenschlager et al., 2022). Our study expands upon these previous findings, showing that phantomless calibration can be applied to healthy volunteers in clinical trial conditions. We also confirmed that the most accurate calibration values were obtained when using the air and aorta as internal materials. Additionally, our study investigated changes across two different timepoints, with scans acquired using two different CT machines, demonstrating robust and broad applicability of this calibration method.

An advantage of QCT imaging of bone is that the resulting scans can be used to create 3D biomechanical models, using finite element (FE) analysis of the vertebra to estimate the bone strength. Patient-specific FE models have long been used to investigate the biomechanical response of bones to loading (Engelke et al., 2016; Schileo and Taddei, 2021). FE has been used increasingly in bones affected by diseases such as osteoporosis (Matsumoto et al., 2009) and different types of cancers including breast, colorectal and renal cell carcinoma (Costa et al., 2019). These models have also been used to study the effect of treatments and have been proven to predict vertebral strength more accurately than DXA in individuals without skeletal diseases (Crawford et al., 2003; Dall’Ara et al., 2012) and with osteoporosis (Imai, 2015). We have recently applied such FE modelling using the ANTELOPE trial dataset reported here, using phantom based calibration to investigate the effects of androgen deprivation therapy on vertebral biomechanics (Gibson et al., 2025). In that study we found that simple BMD changes due to treatment did not capture the full extent of the degradation in vertebral strength evidenced in the FE models. Therefore it remains to be seen whether the similar correlation between phantom and phantomless calibration shown here, and elsewhere (Bartenschlager et al., 2022), will propagate through into resulting predictions of fracture risk and thus further study in this area is required.

## Data Availability

The original contributions presented in the study are included in the article/supplementary material, further inquiries can be directed to the corresponding author.
